# Artificial controlled model of blood circulation system for adhesive evaluation

**DOI:** 10.1038/s41598-017-16814-3

**Published:** 2017-12-01

**Authors:** Sang-Myung Jung, Goo Yong Chung, Hwa Sung Shin

**Affiliations:** Department of Biological Engineering, Inha University, Incheon, 402-751 Korea

## Abstract

Since there are several casualties due to uncontrolled bleeding resulting from simple injury to surgery, effective styptic or vessel adhesives are important; however, their development is limited by the lack of standardized systems to evaluate potential compounds. The current study outlines the development of an aorta styptic evaluation system, comprising of decellularized swine aorta tissue and a heart pump-mimicking system. Although the cells in the swine aorta were removed, the structural stability of the aorta was sustained due to the maintenance of the extracellular matrix. Using a control adhesive, Cyanoacrylate, the developed model was found to have similar adhesive efficacy to intact aorta. The circulatory-mimicking system was designed to mimic the beat rate and strength of blood-flow from the heart, which was necessary to evaluate the adherent efficacy. The decellularized aorta improves instabilities of intact tissues, which occurs on account of storage and origin, thereby allowing for a more standardized system. The system was able to simulate several symptoms of circulation, according to patient age and health, by adjusting pumping frequency and intensity. Therefore, this system can be used as a standardized evaluation system for screening adhesives. Further, it would also evaluate other medical devices, such as stent or medications.

## Introduction

Blood plays an essential role in supplying nutrients and oxygen, and removing waste molecules. The first call of treatment in an accident is to staunch blood loss from injured blood vessels, usually with physical adhesive treatments, such as bandage-pressing or surgery. Biological and chemical adhesive are also available to be used as adjuvants for light injuries and internal bleeding^1–3^. In cases of complex injuries or defects on major vessel, surgical treatment is required, since bandage-pressing method is usually temporary, hard to apply, and ineffective^4^, especially, when main vessels, such as the aorta, which plays a role in sending blood to whole body to maintain organ status, are injured. The aorta can be injured by external causes (e.g., rupture) or internal causes (e.g., hypertension, aneurysm, etc.)^5–8^, which causes trauma or other vascular diseases. Cases of trauma need to be treated within the optimal ‘therapeutic window’, while physical properties that can be used as adjuvant and treating agent during surgery should be identified in cases of vascular disease^9,10^. High blood pressure and heart beat frequency or complex injuries make it difficult to treat wounds using bandage-pressing for extended periods of time. Although adhesive substances are in demand for emergency treatment before surgical intervention, they are not well commercialized^11^.

An ideal adhesive must have low cytotoxicity, appropriate adhesiveness, and mechanical strength. Generally, animal-testing is required to evaluate the ability of the adhesive substance to effectively close a bleeding wound^12,13^. Although, these animal models are well-defined and present appropriate physical (i.e., flow volume, beating rate, and blood pressure) and histological environments (i.e., vessel structure and blood composition) to evaluate adhesive substances, larger animals are required to adequately mimic humans^14,15^. In hemostasis tests, a critical wound in the circulation system can cause animal death, which can lead to ethical problems. When the test is performed in a way that involves critical structures, such as the aorta and arteries, the possibility of error cannot be excluded, which critically affects the survival of the experimental animals. In addition, since the characteristics of animal tissue can vary with age, weight and so on, it is hard to standardize animal models for reliable and reproducible data. Moreover, it is challenging to aptly evaluate injury to the aorta, which is characterized by high blood pressure and active pulsation^16,17^, in models, which brings about issues of reliability and reproducibility of experiments.

**Figure 1:**
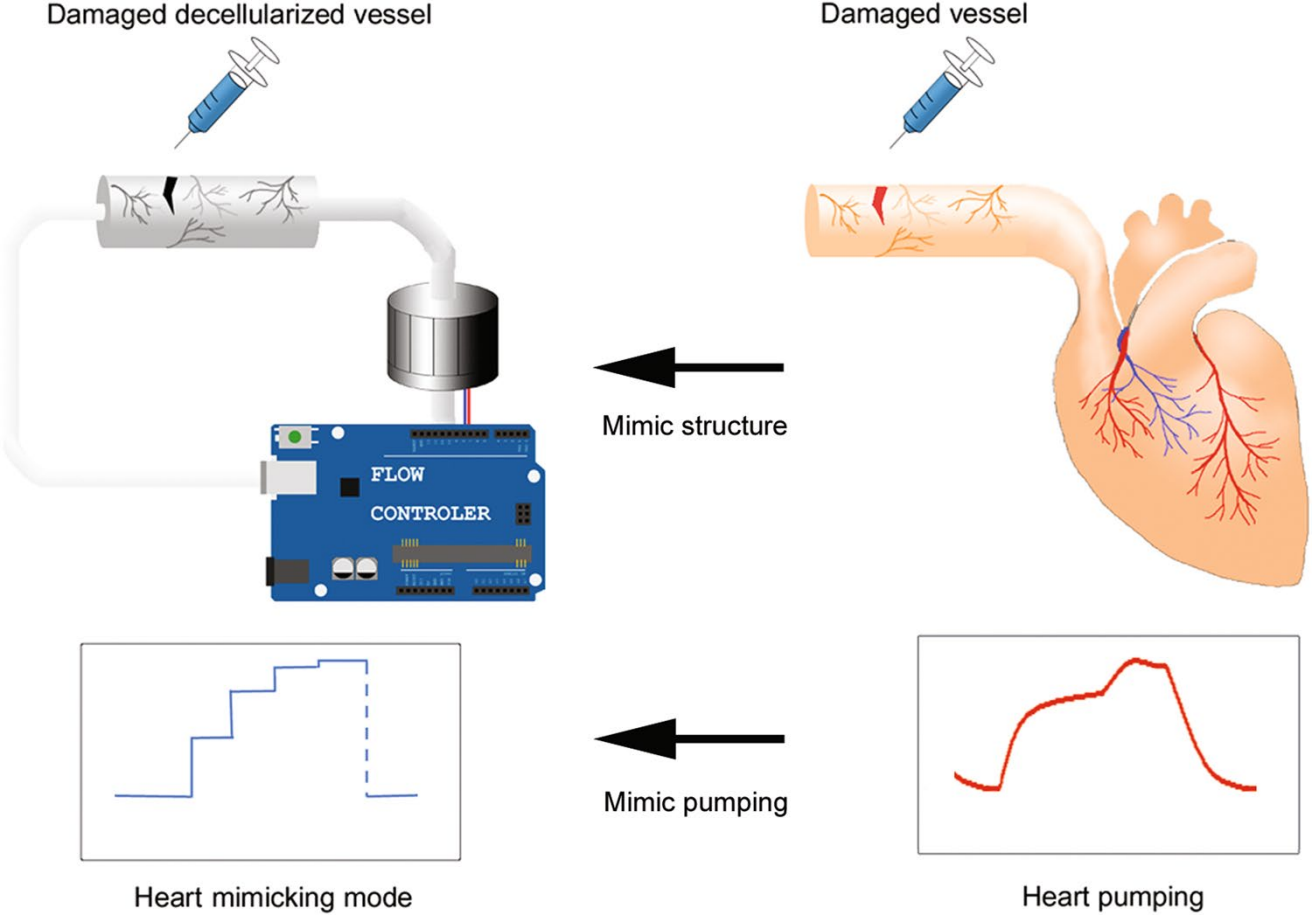
Schematic design of whole circulation system.

In the current work, a bio-tissue/mechanic hybrid system was developed to mimic the injured aorta, with a regular and high frequency of blood flow, and bleeding (Fig. 1). Since several models already exist for evaluating cytotoxicity and safety of adhesives, this system focuses on efficacy test without sacrificing test animals. Our experimental model was superior to a system that does not adequately mimic the injured artery environments, as demonstrated by proof-of-concept test using a rapid emergent adhesive, cyanoacrylate.

## Results

### Confirmation of structural stability of the decellularized vessel

**Figure 2:**
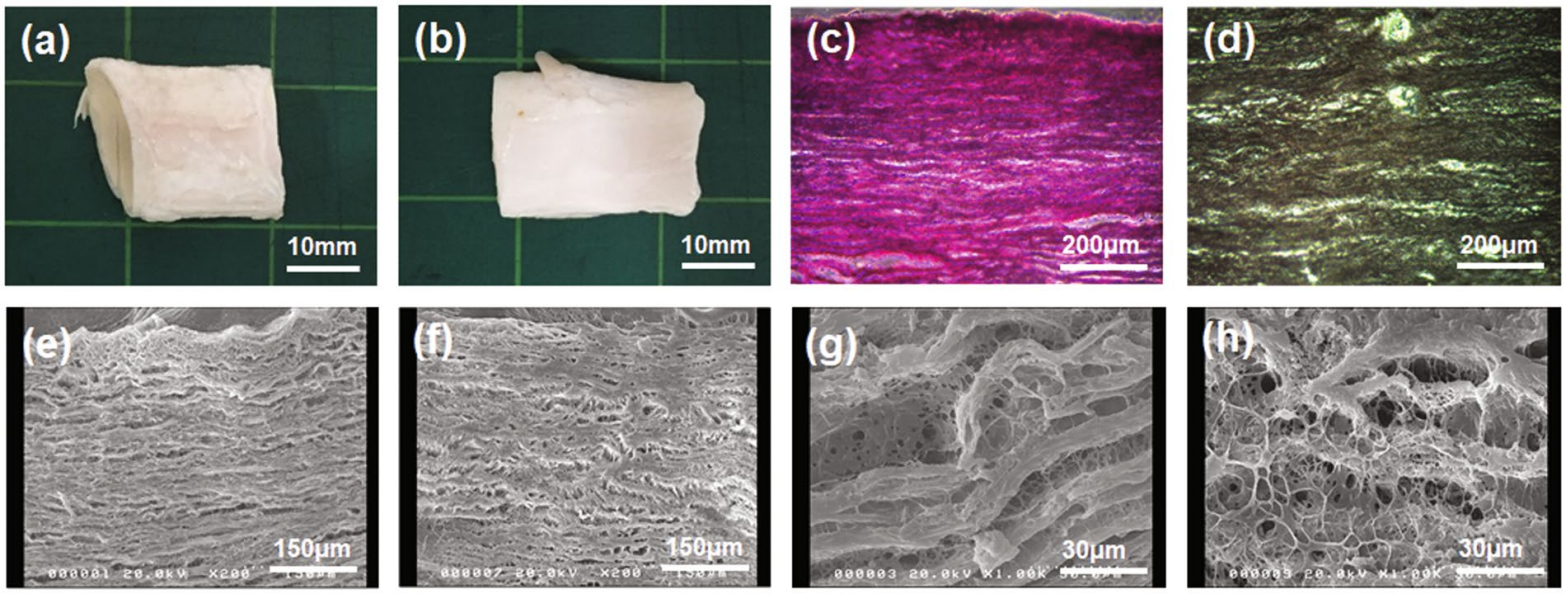
Structural observation of decellularized vessel. Photography of (**a**) cellular vessel and (**b**) decellularized vessel. Cross sectional image of hematoxylin and eosin (H&E) staining of (**c**) cellular vessel and (**d**) decellularized vessel. Scanning electron microscopy (SEM) image of microstructures of the extracellular matrix (ECM) fibrils of cellular vessel at (**e**) low resolution (**g**) and high resolution and decellularized vessel at (**f**) low resolution (**h**) and high resolution.

The decellularized vessel was created using the freeze-thaw method. The vessel maintained its morphology, after 10 repeated freeze/thaw cycles, with no observed additional denaturation or collapse at the vessel (Fig. 2a,b). The results of the hematoxylin and eosin (H&E) staining also showed that the cytosol stained by eosin was removed from the decellularized sample, compared to the original sample, but both samples had abundant extracellular matrix (ECM) and both ECM structure were observed well at microscopy images (Fig. 2c,d) and SEM images (Fig. 2e–h). Except decellularized vessel was shown more porosity, the microscopic images confirmed that the layer-by-layer structured ECM was not apparently different between non-decellularized (Fig. 2c) and decellularized vessel (Fig. 2d). The difference of porosity not effective to collapse of vessel is formed by removal of cells in decellularized vessel. In SEM images, non-decellularized vessel had dense ECM layer (Fig. 2e,g) and decellularized one maintained a layer-layer structure of ECM well (Fig. 2f,h). Thus, although cells in the vessel were removed, the ECM was well maintained even after decellularization.

### The aorta vessel retained its mechanical strength after decellularization

**Figure 3:**
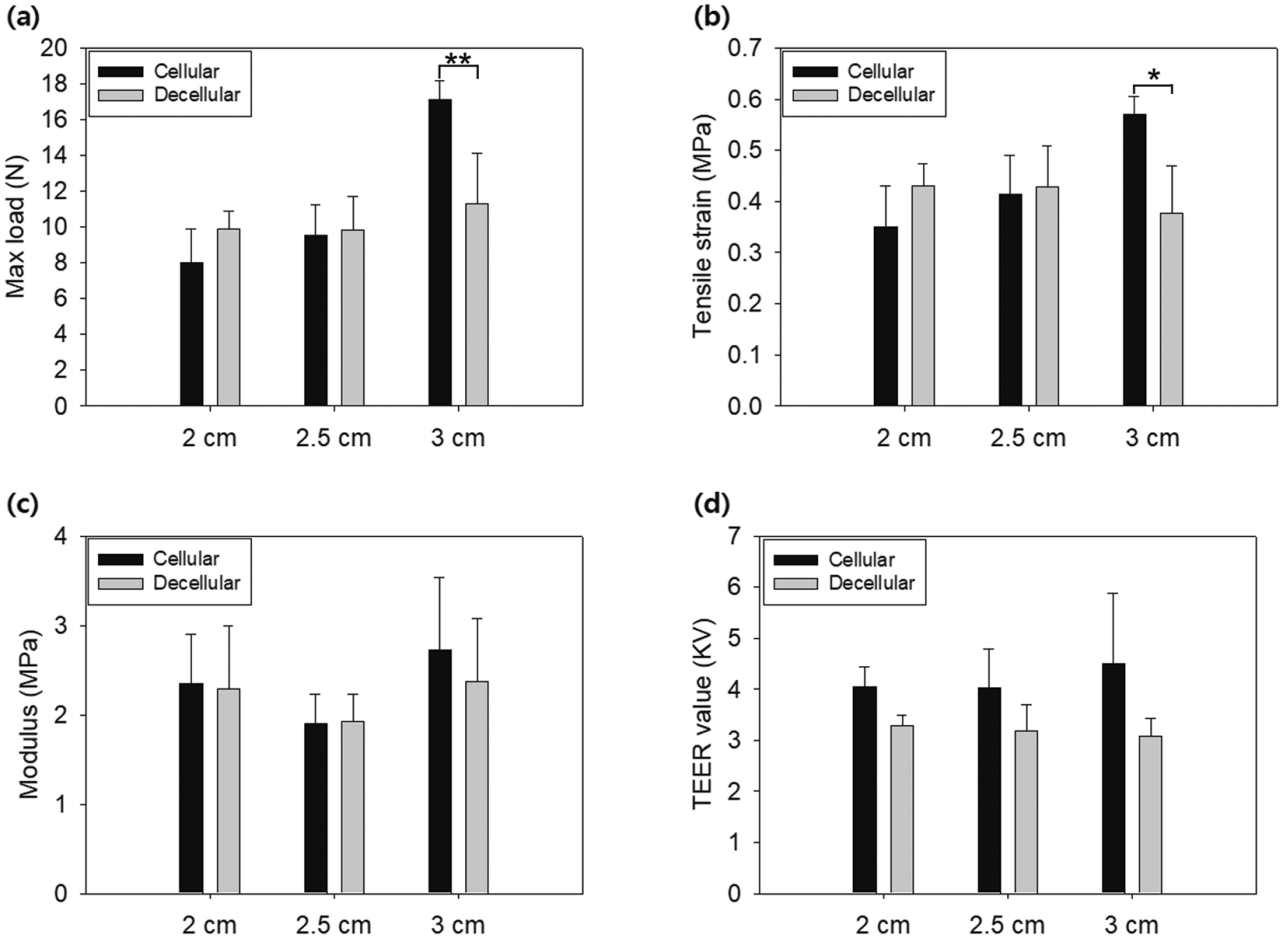
Mechanical characteristics of vessels by treatment. (**a**) Maximum load of cellular vessel and decellularized vessel. (**b**) Tensile strain and (**c**) Young’s modulus of cellular vessel and decellularized vessels. (**d**) Transepithelial electrical resistance (TEER) of the cellular and decellularized vessels. *P < 0.05; **P < 0.01 (Student’s t-test). Error bars indicate standard deviation.

Mechanical strength was compared between original and decellularized vessels with three different diameters in terms of physical characteristics: maximum load, tensile strain, and Young’s modulus. The maximum load was not significantly different between samples that were 2 cm and 2.5 cm in diameter, although the decellularized vessel with 3 cm, which are closer to the heart’s ventricles, showed significantly lower value (Fig. 3). This position difference made 3 cm vessel thicker than the other vessels. For this reason, the original 3 cm-thick vessels have a higher maximum load than the other vessels before decellularization. However, decellularization removed much more cells from the vessels of 3 cm diameter compared with the small sized ones, which is thought to lead to less integrity of ECM in 3 cm-thick vessels. In short, much more decellularization resulted in much less integrity of ECM, resulting in a sharp drop in mechanical strength in 3 cm-thick vessels compared with the small sized vessels.

The tensile strain and Young’s modulus were also not significantly different between cellular and decellularized samples, at any diameter. Tensile strain and Young’s modulus are influenced dominantly by GAGs. GAGs functionalize protein to proteoglycan and possibly the formation of a collagen fiber network with increased interstitial spaces and porosity modulus^18^. The GAGs can be removed easily by decellularization using detergents, but in the research, we used freeze-thaw method without detergents. For the reason, tensile strain and Young’s modulus were not changed significantly when the maximum load was decreased after decellularization. The porosity of vessel was quantitatively measured by trans-epithelial electrical resistance (TEER). Diameter of vessel did not make any significant differences in TEER values for both decellularized and non-decellularized vessels. However, TEER value was lower in decellularized vessels than in non-decellularized vessel (Fig. 3d). This implied that vessel lost its t-junction during cell removal by decellularization, even though the layer-by-layer structure did not look significantly different from that of non-decellularized vessel.

### Development of heart-mimicking circulation system (HMCS)

**Figure 4:**
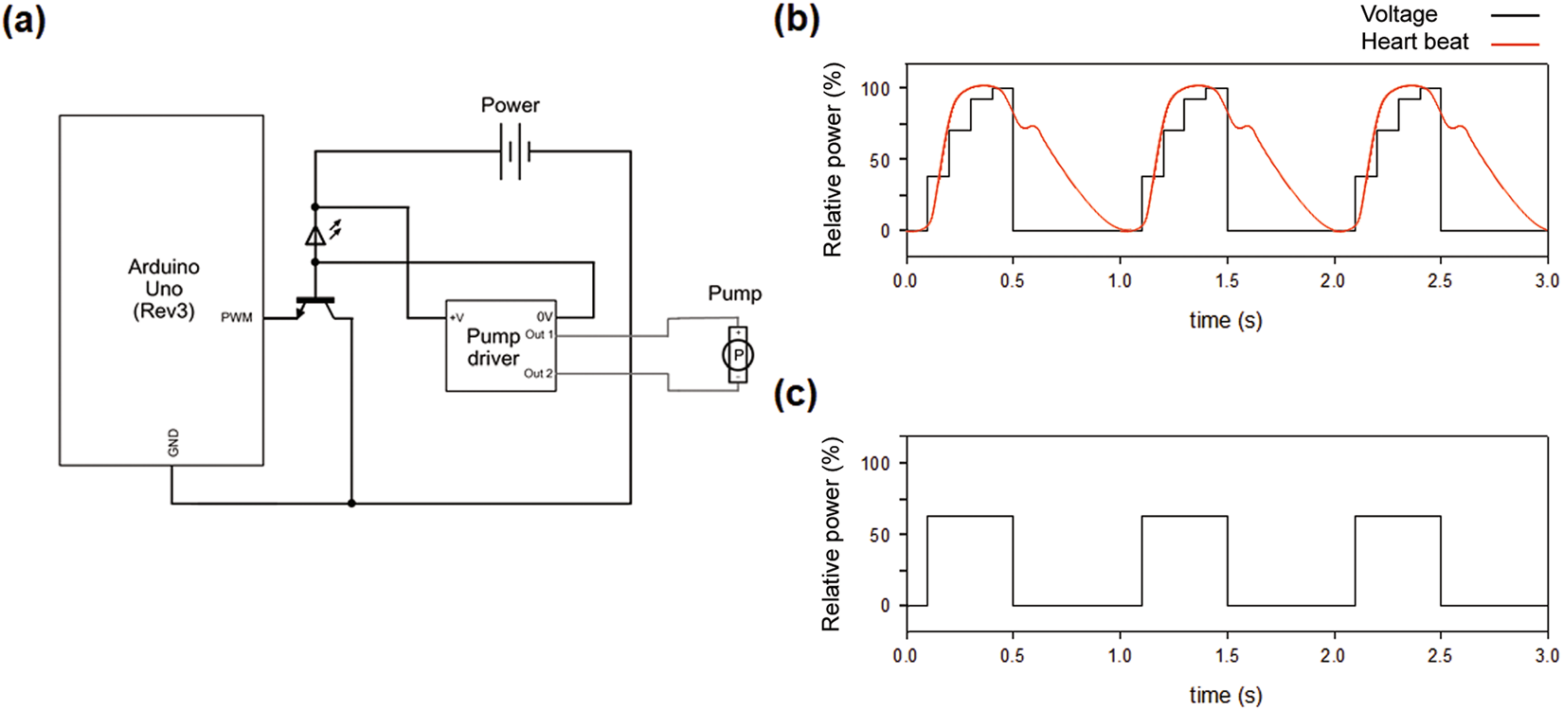
Schematic of the circulation system and its operation. (**a**) Circuit diagram of the system from controller to pump. The operating patterns of the circulation system was as follows: *in vivo* heart, input signal, and output signal, of (**b**) gradient type and (**c**) bang-bang type operation.

The system was assembled and operated continuously to mimic heart beating and pumping (Fig. 1). As shown in Fig. 4a, the pump was controlled by an Arduino control box. The speed of the pump depended on the strength of the voltage applied from the power source. The Arduino sent an on/off signal to the transistor and controlled the voltage from power source to pump. The pump aspirated and emitted media to the decellularized vessel and then the media fed back to the pump.

The pumping frequency and volumetric flow rate were used to mimic the hydrodynamic environment of an aortic site connected to the heart. In the control box, voltage was adjusted to regulate ON/OFF time and be responsible for regulating volumetric flow rate out of the pump. Heart beat profile has monotonous shape at systolic phase and the blood is spewed out. After systolic phase, there presents diastole phase where pressure drops slowly and blood is not spewed out even if there is residual pressure in heart valve. In case of gradient type, each 38.4, 70.5 92.5, 100% voltage/maximum voltage was transmitted to pump at 0.1 s interval and it took rest period for 0.6 s. The bang-bang type transmitted 62.0% voltage/maximum voltage for 0.4 s and rest for 0.6 s. As a demonstration, heart beats at 60–100 beats/min, sending approximately 17 L/min of blood to the aorta, and the heart pumps 70 ml of blood during each systole phase, which usually has duration of 0.4 s^19^. In order to mimic blood flow, HMCS was manipulated to pump at 60 times/min, sending 70 ml/s of media to the aorta vessel. Finally, the heart pumping and the flow blood pattern were mimicked in the similar way to those of heart (Fig. 4b).

### Structural stability of the decellularized vessel connected to HMCS

**Figure 5:**
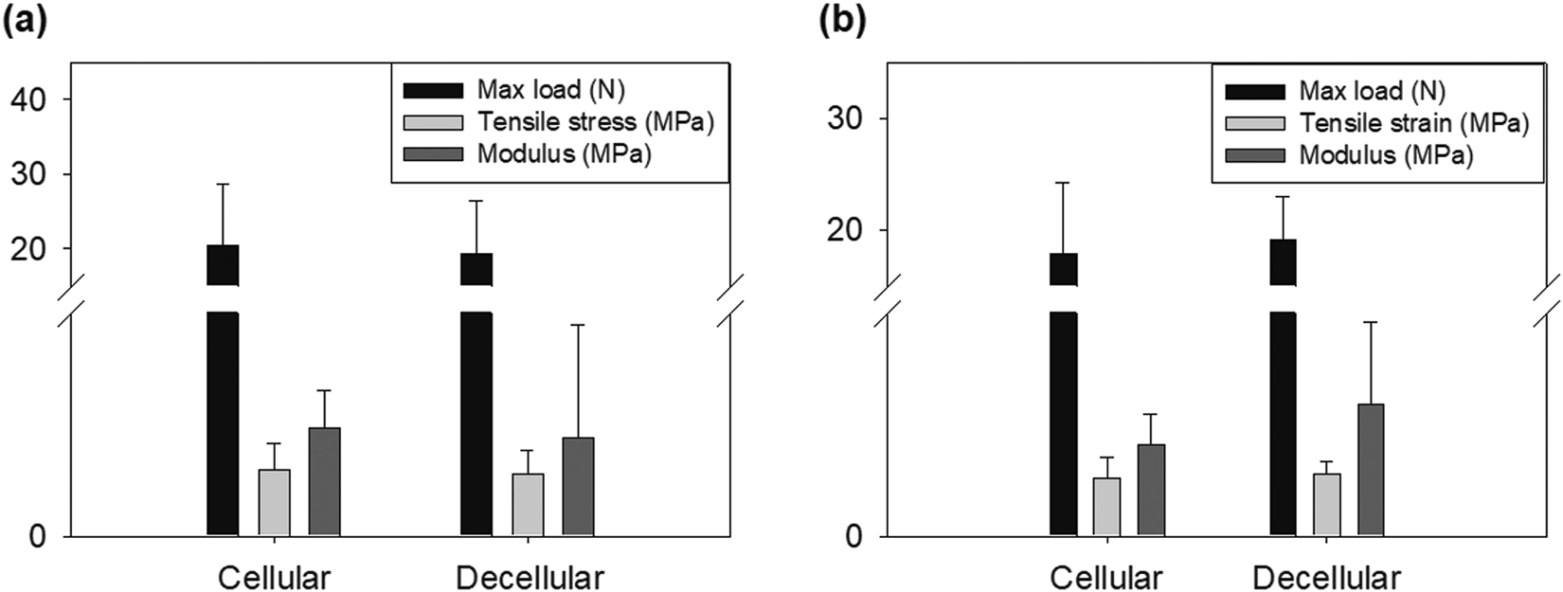
Universal testing machine data (UTM) data for stress test with alternative vessels, i.e., cellular and decellularized vessels. The UTM data (maximum load, tensile strain and Young’s modulus) of (a)stress test and (b)adhesive test using cellular vessel and decellularized vessel. *P < 0.05; **P < 0.01 (Student’s t-test). Error bars indicate standard deviation.

The decellularized vessel connected to HMCS was evaluated of its structural stability after exposed to pressure of flow emitted from HMCS. As seen in Fig. 5a, the mechanical strength of decellularized vessel (Max load: 19.40 ± 7.12 N, tensile strain: 0.84 ± 0.30 MPa, modulus: 1.32 ± 1.51 MPa) were slightly decreased than those of cellular vessel (Max load: 20.42 ± 8.26 N, tensile strain: 0.89 ± 0.36 MPa, modulus: 1.46 ± 0.50 MPa) but there were no significant differences through statistical comparison.

### Usefulness of the decellularized vessel as a test model for an adhesive

To verify if the decellularized vessel is useful for efficacy testing of adhesive, both decellularized and original vessels took 1 cm length rupture and then was treated with adhesive; cyanoacrylate. The testing system must present leaking pressure after rupture to evaluate adhesives in a similar situation with aorta. The pressure applied to the rupture of 2 cm or more was lowered and the fluid was not ejected to the rupture area. But, 1 cm and 1.5 cm rupture expressed significant flow ejection, not showing much difference in both (data not shown). For the reasons, 1 cm length rupture was selected to demonstrate the effect of leaking pressure on adhesive efficacy. After adhesive treatment, the vessel was followed by exposure to flow pressure from HMCS, which resembled frequency and volumetric flow rate of heart-pumping. No significant differences in mechanical strength were detected between them. Original vessel showed 17.90 ± 6.30 N at max load, 0.78 ± 0.27 MPa at tensile strain and 1.23 ± 0.42 MPa at modulus. Decellularized vessel showed 19.10 ± 3.89 N at max load, 0.83 ± 0.17 MPa at tensile strain and 1.76 ± 1.11 MPa at modulus. There were no significant differences of each parameter between the samples by student t-test (Fig. 5b).

### Significance of the HMCS for a test model of adhesive

**Figure 6:**
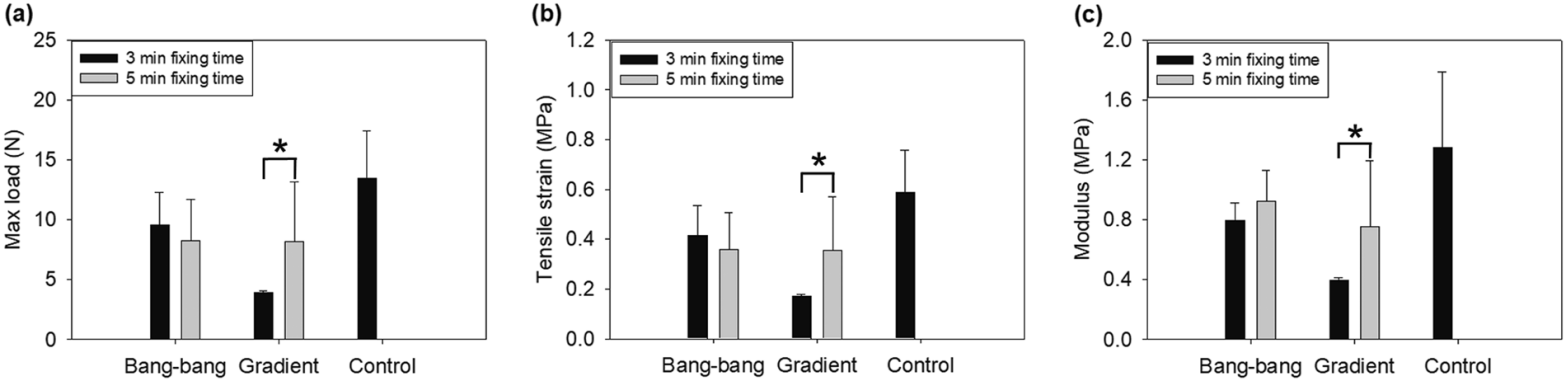
Universal testing machine (UTM) data of adhesive evaluation with gradient types and bang-bang type system, with different fixing times and operation types as outlined in Table 1. (**a**) Maximum load value of vessel for each test condition. (**b**) Tensile strain and (**c**) Young’s modulus for each test condition. *P < 0.05; **P < 0.01 (Student’s t-test). Error bars indicate standard deviation.

Flow into vessel is a major momentum to mechanical stress against vessel wall and thus is an essential point that must be overcome in adhesives. The volumetric flow of heart is 70 ml/beat but frequency may not be consistent because of systolic trend of heart, leading to each different hydrodynamic pressure with respect to a flow pattern. For this reason, adherent and fixing efficacy needs to be evaluated according to a flow pattern. In this research, the two pumping types; gradient and bang-bang types, were compared for cyanoacrylate adhesiveness. Gradient type showed different mechanical properties according to the time of treatment with the adhesive. The maximum load was significantly decreased, compared with that of bang-bang type with a treatment time of 3 min. The maximum load was recovered to just 63.9% of non-torn site. When the rupture was torn again during operation, leaks were observed, suggesting that the adhered site was not uniformly maintained. Further, the tensile stress at maximum load and modulus were also decreased. The adhered site was too weak and could not resist stress before the other non-damaged area was fully extended. In addition, cyanoacrylate hardens during polymerization, making the treated site inflexible. However, it took more than 5 min of treatment, before the mechanical characteristics were recovered to a similar level as the bang-bang vessel. Adhesive-treated vessel for 5 min showed no significant differences compared with no-torn vessel and re-tear of the rupture site was also not observed (Fig. 6, Table 1). All these results implied that pumping frequency and strength is critical to test adhesive efficacy.

**Table 1:** Test conditions and numerical values of the mechanical characteristics of each condition (Fig. 6).

Type	Fixing time	Max load (N)	Tensile strain at max load (MPa)	Modulus (MPa)
Bang-bang	3 min	9.59 ± 2.71	0.42 ± 0.12	0.79 ± 0.12
	5 min	8.28 ± 3.38	0.36 ± 0.15	0.93 ± 0.21
Gradient	3 min	3.89 ± 0.21	0.17 ± 0.018	0.40 ± 0.016
	5 min	8.15 ± 4.99	0.35 ± 0.28	0.76 ± 0.44
Control	—	12.76 ± 10.36	0.55 ± 0.45	1.23 ± 1.18

## Discussion

A standard evaluation system to develop an adhesive for injured aorta requires both standardized aorta vessel and beating flow system to mimic blood pumping from the heart. The efficacy of the incorporated both system must also be assessed, prior to its use to evaluate candidate adhesives.

The use of aorta vessel that is regenerated using a standard tissue engineering protocol would be best in such a system. However, the development of an artificial aorta by tissue regeneration is limited due to the specificity of its structure, i.e., blood vessels are composed of the three following layers; intima, media, and adventita^20^. The media layer consists of smooth muscle cells and ECM (typically collagen and elastin) that is so densely packed, it dominantly affects the mechanical characteristics of blood vessel. Further, the 4-mm thickness of the media layer is also too hard to mimic by tissue engineering. Thus, use of a real aorta is an alternative to address this problem. Swine aorta is one of the best choices since swine tissue is similar to humans. However, since the characteristics of tissue can vary with age, and the cells in the tissue are difficult to standardize, using the tissue in its intact form would increase statistical uncertainly in the evaluation. In addition, the vessel was already densely packed by the ECM of endothelial cells and smooth muscle cells of the intima layer and external layer, respectively^21,22^, and these cells are a risk factor for tissue collapse due to ribosomes or several enzymes, such as collagenase that are critical to the external layer, which is collagen-rich and dominates to tissue structure^23^. Further, since the adherent material is applied to outer surface of the tissue, it is also important to evaluate the effect of adhesives on the ECM structure. For these reasons, decellularized aorta, which maintains its intact ECM structure, was proposed as the standardized tissue system.

As demonstrated by its structural and mechanical properties (Figs 2 and 3), the decellularized swine aorta represented structural integrity and mechanical strength comparable to that of intact tissue, although the t-junction was removed. The maximum load is the major characteristic that gives rise to resistance against mechanical stress derived from fluid flow^24^. It affects system robustness, required a stronger pump to mimic heart pressure. The tensile strain and modulus affects the strain and movement of the tissue layer^25^. The lateral expansion by the pulse of fluid is changed by these physical properties^26^. However, since the physical properties of the decellularized sample were not changed significantly; this meant that the vessel beating or expansion would be well-mimicked in the current model. These results implied that ECM was mainly configured by structural proteins, such as collagen fibrils and elastin, which are dominant contributors to strength among tissue^27^, and were not destroyed or denatured by decellularization^28^.

These results corroborated with previous findings, demonstrating that decellularization had no effect on the mechanical characteristics of the media layer and that removing cells enhanced its storage ability^29,30^. Meanwhile, t-junction seals the gap between cells and separates inner/outer layers^31^, but it was concluded not to significantly affect mechanical strength compared with the layer-by-layer structured ECM, judging from the fact that the decreased TEER in decellularized tissue was not consistent with the non-varying mechanical strength of the vessel (Fig. 3). All these results implied that the major factor of tissue strength was ECM, rather than the t-junction. Since decellularization did not affect the physical properties of vessel tissue, again these results highlighted that the current system was adequate in mimicking the circulation system.

The *in vivo*-like pulsation and blood flow system was realized by controlling a pump with a pre-determined schedule. The beating frequency and strength of blood flow varies among people, according to their age and health. However, the use of an Arduino controller coupled with actuator pump, allowed pumping to reflect the parameters in reference to healthy and adult^32^. As demonstrated in Fig. 4, the blood flow emitting from the heart was successfully realized through this system. It was easy to change the frequency and strength of the flood, using the mechanical pump and controller, and these could be reliably reproduced. Thus, the system developed in the current study could be regarded as a standardized HMCS. Furthermore, the decellularized vessel could retain its mechanical strength similar to that of original vessel even after exposed to flow pressure out of HMCS. Therefore, the hybrid system securely modeled an *in vivo* aorta vessel. Although the decellularized and cellular samples had different TEER values as shown in Fig. 3a, it did not affect the mechanical resistance against the pressure derived from the pump, but the retained ECM of the decellularized vessel guaranteed its resistance to flow pressure.

Two rationales were applied to prove the efficacy of the developed system. The first requirement was that the model adhesive efficiency must be similar, in both intact and decellularized aortas, so that the decellularized aorta could be validated as a modeling standard tissue. To investigate this, the pumping condition was fixed in both cases, mimicking the blood flow emitting from heart. As illustrated in Fig. 5, application of cyanoacrylate showed similar mechanical properties in both vessels, implying that decellularized aorta could be useful in evaluating adhesives, overcoming the challenge of standardizing the use of intact aorta. As shown in the results, mechanical strength was not critically different between original and decellularized tissue (Figs 5a and b). As shown in Fig. 5a, the physical properties of each vessel (original and decellularized vessel) were the same in intact form without application of adhesive. Also, the mechanical property of each vessel was not significantly different after treatment of the adhesive (Fig. 5b). Therefore, the porosity of the decellularization did not significantly affect the performance of the system. For the reason, we can suggest that the decellularization removes cytosol and interaction between cytosol and collagen fibril, then maximum load is decreased but collagen fibril and its network still remains in intact form and are dominant to maintain mechanical strength in tissue. The second requirement was that the efficiency of an adhesive to decellularized aorta must be dependent on pumping frequency and strength of HMCS. To investigate this, we compared the efficiency of cyanoacrylate in the heart pumping-mimicking system and bang-bang type pumping system. The mechanical properties were weaker in HMCS, which implied that the pumping frequency and strength affected the adhesive efficacy. This finding highlighted and validated the importance of evaluating adhesives using HMCS.

Therefore, HMCS developed in the current study met all the requirements to evaluate an adhesive for the injured aorta. Further, the system and evaluation protocol was standardized. There are several other advantages of this system too. In cases requiring such a formalized system, decellularization saves resources as the aorta section is easier to prepare. Further, there is strength in having a standardized system, since results are replicable and comparable. Moreover, this system minimizes the use of experimental animals, overcoming the ethical challenges of conducting preclinical studies. HMCS that was developed in the current study has the potential to allow researchers to conduct more accurate preclinical studies.

Adhesive is an effective treatment to blood vessel injury, but its development is limited by the lack of an appropriate evaluation system to assess its efficacy in hard-to-accessible vessels, such as the aorta. In the current study, a standardized and biomimetic system was developed to replace animal-testing models in evaluating circulation-system drug. HMCS mimicked the parameters of an *in vivo* circulation system, such as pumping frequency and volumetric flow rate, which were easily and reproducibly adjusted using an automatic pump system. Further, we conducted a proof-of-concept experiment to demonstrate the efficacy of HMCS in evaluating the adhesive properties of cyanoacrylate. Overall, we validated the use of the developed system in evaluating novel adhesives, and demonstrated that it can help save resources and overcome the ethical challenges of using animals during preclinical studies.

## Materials and Methods

### Vessel part formation: Decellularization

For organizing a vessel part of circulation system, we decellularized a swine aorta from a butcher in Incheon, South Korea. The vessel was cut to 3 cm in length, and washed with ethanol to remove fat and contaminants. Before freezing, ethanol was exchanged with distilled water. The vessel was frozen using liquid nitrogen for 30 s, thawed fully in ambient temperature for 10 min. The freeze-thaw routine was repeated 10 times. The distilled water contained the residue was changed and the decellularized sample was stored at −20 °C.

### Sectional structure observation

The cross-sectional morphology was observed to confirm vessel decellularization. The swine vessel was cut into an area of 1 × 1 cm^2^, and immersed in 30% and 60% sucrose solutions, for 4 h, each. After the treatment, optimal cutting temperature compound was poured on the vessel to make a block and the block was sliced at a thickness of 40 µm. Cell nuclei were stained using 4′,6-diamidino-2-phenylindole (DAPI) and tissue histology was observed with hematoxylin and eosin staining.

### Vessel decellularization: Analysis using an universal testing machine

Universal testing machine (UTM; Instron, MA, USA) was used to confirm differences in physical characteristics (e.g., maximum load, tensile strain, and Young’s modulus) of the vessel following decellularization. The vessel was cut in the horizontal direction into equal areas of 1 × 3 cm^2^. The pieces of vessel were steadily elongated at 2 mm/min by UTM and physical characteristic values were recorded.

### Decellularized vessel trans-epithelial electrical resistance

TEER was measured to confirm the intercellular junctions by decellularization. The vessel, from which an area of 1 × 1 cm^2^ was cut, was measured with a TEER sensor. Measurements were replicated at least three times per sample.

### Circulation system formation

The following items were used to form the system: water pump, silicon tube, connector, and an Arduino control box. The decellularized blood vessel was placed between connectors, which linked it with the motor, which controlled the flow and frequency. The pump, which was operated by the Arduino control box, pumped 60 times/min. A total of 70 mL of media was ejected as once during systolic work, which was 0.4 s in duration.

### Application of decellularized vessel supplemented to cellular vessel

The decellularized vessel was compared with a cellular vessel. The samples were installed into the system and the system was operated for 30 min with distilled water at 37 C. The samples were harvested and their physical characteristics were evaluated using UTM.

### Adhesive evaluation using a tissue adhesive

We tested the effect of cyanoacrylate (Sigma-Aldrich, MO, USA), a major tissue adhesive, on the circulation model system to confirm that the system could be used to evaluate potential candidates for vessel adhesion. After operating the circulation system, a 1-cm-sized cut was made on the vessel. Cyanoacrylate was applied to the cut, and differences in leaking volume through the cut and flow rate in the vessels were measured. The vessel was harvested and its physical characteristics were measured using UTM. We evaluated the potential of cyanoacrylate as a vessel adhesive based on these results.

### Statistical analysis

All experiments were repeated at least 3 times. The asterisks were added when the statistical differences were observed by a Student’s t-test as follows: *p < 0.05; **p < 0.01; and ***p < 0.001.

## Electronic supplementary material

Supplementary Video S1

Supplementary information
