# Author Correction: Artificial controlled model of blood circulation system for adhesive evaluation

**DOI:** 10.1038/s41598-018-24839-5

**Published:** 2018-04-19

**Authors:** Sang-Myung Jung, Goo Yong Chung, Hwa Sung Shin

**Affiliations:** Department of Biological Engineering, Inha University, Incheon, 402-751 Korea

Correction to: *Scientific Reports* 10.1038/s41598-017-16814-3, published online 01 December 2017

This Article contains errors in Reference 3 which was incorrectly given as:

Wojciech, W., Agnieszka, S.-P., Magdalena, B. & Tomasz, G. Experimental Comparison of Efficiency of First Aid Dressings in Burning White Phosphorus on Bacon Model. *Med Sci Monit*. **21**, 2361–2366 (2015).

The correct reference is listed below:

Witkowski, W., Surowiecka-Pastewka, A., Biesaga, M., Gierczak, T. Experimental Comparison of Efficiency of First Aid Dressings in Burning White Phosphorus on Bacon Model. *Med Sci Monit*. **21**, 2361–2366 (2015).

