# Supplementary material for: Artificial controlled model of blood circulation system for adhesive evaluation
Source: Sci Rep. 2017 Dec 1;7:16720. doi: 10.1038/s41598-017-16814-3 (PMC5711803; doi:10.1038/s41598-017-16814-3)
Supplement: Supplementary file 2 — Supplementary information [file 41598_2017_16814_MOESM2_ESM.pdf]

Artificial controlled model of blood circulation system for adhesive evaluation

Sang-Myung Jung[1], Goo Yong Chung[1], Hwa Sung Shin[1, *]

[1]Department of Biological Engineering, Inha University, Incheon, 402-751, Korea

*Corresponding author.

Hwa Sung Shin, hsshin@inha.ac.kr, Tel: 82-32-860-9221, Fax: 82-32-872-4046

This file includes:

1 figure, 2 tables and 1 video which are supporting our research.

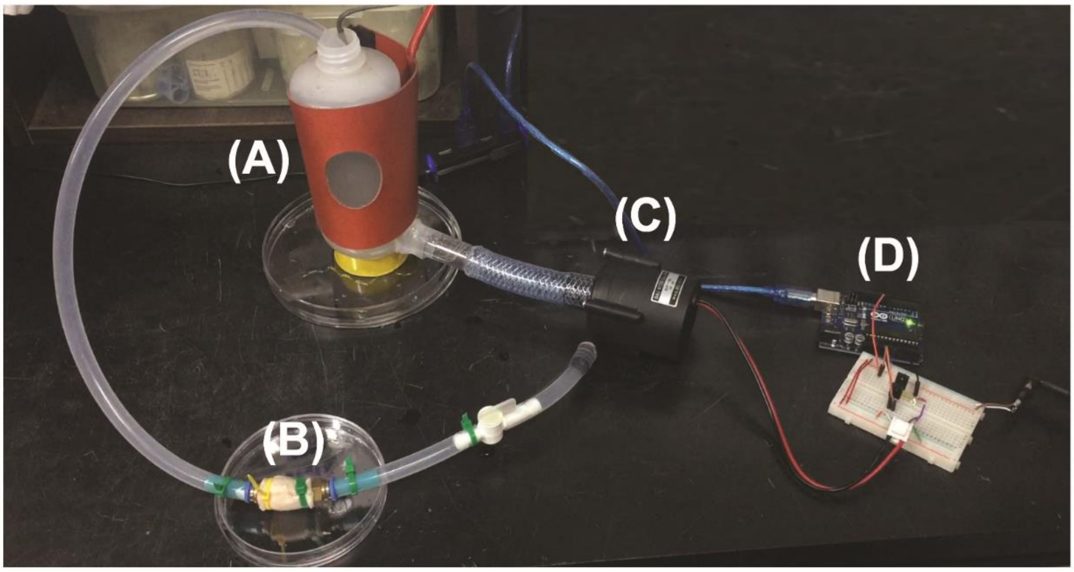

Supplementary Figure S1 Whole system of heart-mimicking circulation system (HMCS). (A) Reservoir of fluid, (B) Vessel part, (C) Pump and (D) Controller and circuit.

Supplementary Table S1 Set and measured values of relative voltages supplied to the pump during system operation at each type. The voltage was supplied for 0-0.4s and it stopped for 0.5-1.0 s. This 1 s cycle was operated repeatedly on system operation.

	**Time (s)**	**0**	**0.1**	**0.2**	**0.3**	**0.4**
**Gradient type**	**Set value (%)**	0	38.4	70.5	92.5	100
	**Measured value (%)**	0	38.29±0.09	70.51±0.03	92.47±0.08	100.01±0.05
**Bang-bang type**	**Set value (%)**	0	62	62	62	62
	**Measured value (%)**	0	61.97±0.11	61.99±0.12	62.00±0.12	61.92±0.09

Supplementary Table S2 Set and measured values of spitting fluid volume from pump at a cycle at each type. The pump spitted out 70 ml per a cycle repeatedly.

	**Set value (ml/cycle)**	**Measured value (ml/cycle)**
**Gradient type**	70	70.14±1.08
**Bang-bang type**	70	70.03±0.92

Supplementary Video S1 Demonstration of beating on vessel at each condition (intact vessel, ruptured vessel, vessel with completely fixing and with incompletely fixing) using the system.
